# Health Insurance Payment for Telehealth Services: Scoping Review and Narrative Synthesis

**DOI:** 10.2196/56699

**Published:** 2024-12-09

**Authors:** Qingqing Li, Feng Cheng, Huatang Zeng, Junfang Xu

**Affiliations:** 1 School of Public Health, Second Affiliated Hospital Zhejiang University School of Medicine Hangzhou, China China; 2 Vanke School of Public Health, Institute for Healthy China Tsinghua University Beijing China; 3 Shenzhen Health Development Research and Data Management Center Shenzhen, China China

**Keywords:** telehealth, internet-based health care, reimbursement, health insurance payment, health care, scoping review, narrative synthesis, mobile phone

## Abstract

**Background:**

As telehealth services have demonstrated significant advantages in providing qualified and accessible care, health insurance payments for telehealth services have been issued by various countries. However, the optimization of health insurance payments for telehealth services remains uncertain.

**Objective:**

We conducted a scoping review of the current situation regarding health insurance payments for telehealth services, with the aim of providing evidence to enhance policies related to health insurance payments for such services.

**Methods:**

This scoping review was conducted by comprehensively retrieving data from 6 electronic bibliographic databases from inception to October 2023. The databases included China National Knowledge Infrastructure, Wan Fang, Weipu, Web of Science, PubMed, and Embase, following the PRISMA-ScR (Preferred Reporting Items for Systematic Reviews and Meta-Analyses extension for Scoping Reviews) guidelines. Two authors independently assessed search results, extracted data, and evaluated the quality of the included studies using the Critical Appraisal Skills Programme checklist. After the initial screening of titles and abstracts, full texts were obtained and examined. The data regarding the first author, date of publication, country, type of telehealth services introduced in health insurance, health insurance reimbursement providers, reimbursement standards for telehealth (eg, the condition for the reimbursement and reimbursement rate), and key findings of studies were extracted and analyzed. Moreover, we also conducted a narrative synthesis to summarize and report the findings.

**Results:**

A total of 7232 papers were retrieved. Following quality assessment, 23 papers were finally included, with the covered countries including China, the United States, Australia, France, Japan, the United Kingdom, and Germany. The forms of the services vary across different regions, mainly including consultation services, medical monitoring services, mental health services, health education services, and other services. Payment standards are generally categorized into fee-for-service and global budget payment, with clear health insurance payment quotas or proportions and certain restrictions (eg, specifying the location of medical visits and setting the limitation on distance from home to hospitals). The paying entities for health insurance payment include national health insurance and commercial health insurance. In addition, there are 2 kinds of reimbursement rates—a comparable rate for both telehealth and in-person health care services, and a lower rate for telehealth services compared to in-person health care services.

**Conclusions:**

To enhance the accessibility of telehealth services through health insurance payment, it is crucial to further refine the design of health insurance payment for telehealth and strengthen the supervision of services quality, bridging the gap between telehealth and in-person health care services. Additionally, this review did not include studies from all countries, and we recommend that future reviews should include a broader range of countries to provide a more comprehensive view of global telehealth insurance systems.

## Introduction

Telehealth was first proposed by the World Health Organization (WHO) in the 1970s and was defined as the use of video and communication technologies by medical practitioners for the prevention, diagnosis, and treatment of diseases, as well as health counseling, with the aim of promoting the health of individuals and the community. Common forms include telehealth monitoring, telehealth education, telenursing, teleconsultation, and telecare [1]. In 2022, the global telehealth market was valued at approximately US $87.9 billion. By 2023, this valuation had increased to around US $120.4 billion. The market is projected to grow to about US $98.5 billion by 2028, with a compound annual growth rate of 23.2% [2]. Telehealth services have multiple advantages. First, internet technology breaks through the limitations of time and geography, promoting the decentralization of high-quality medical resources to grassroots, remote, and impoverished areas. This facilitates timely and high-quality medical services for patients, addressing issues arising from the uneven distribution of national medical resources, which is significantly important in promoting the fairness of medical resources. Second, it further enhances the mobility of medical resources by leveraging digitalization and informatization. This facilitates a more flexible and efficient circulation of medical resources by promoting the sharing of medical technology knowledge. Third, it meets patients’ medical care needs with greater convenience and at a lower cost by providing them with medical services without long-term travel. Especially during the COVID-19 pandemic, the scale of telehealth services has expanded significantly considering the implementation of alleviating the need for face-to-face contact in person (eg, home isolation and lockdowns) to prevent the transmission of COVID-19 [3]. Telehealth services possess several key advantages that enable them to respond actively to sudden public health emergencies (PHE). For example, they can meet the demand for timely and professional medical care of patients while diminishing the risk of virus cross-infection [4]. In 2020, telehealth consultations in hospitals affiliated with China’s National Health Commission increased 17-fold compared to the same period in 2019. In the United States, the number of telehealth visits surged by 63 times in 2020 [5]. In the United Kingdom, the usage of video consultations in Scotland experienced a 1000% growth in a 2-week period in March 2020 [6]. Additionally, in Australia, the proportion of consultations conducted through videoconferencing increased from 0.2% in February 2020 to 35% in April 2020 [7].

With the widespread broader adoption of telehealth during the COVID-19 pandemic, public expectations of these services are evolving. Many countries are seizing this opportunity to emphasize the pivotal role of telehealth in the future of health care delivery. This is evidenced by initiatives like expanding internet health care infrastructure, forging partnerships with communication technology companies, diversifying service offerings (eg, telehealth for chronic diseases), and expanding the range of services (eg, telemonitoring and management of chronic diseases), as well as incorporating telehealth services into the reimbursement scope of health insurance.

Different health care systems have been implemented by various countries based on their management capabilities and market needs. The United Kingdom and Australia exemplify the national health insurance model, characterized by government funding and universal coverage. In contrast, Germany, France, and China represent the social health insurance model, where government-regulated funds provide coverage, typically funded through a combination of employer and employee contributions. The United States represents the commercial insurance model, where health care coverage is primarily provided by private insurers. In the process of advancing the implementation of telehealth services, the matter of health insurance payment has been recognized as an obstacle [8-11]. Health insurance payment serves as a compensatory mechanism, empowering more patients to afford the expenses associated with telehealth services. This is particularly crucial for individuals with limited financial means or those in need of long-term medical services. Such support not only stimulates the demand for telehealth services but also fosters an equitable distribution of health care resources, enhancing the efficiency of their use. However, comprehensive evaluations of health insurance payment policies for telehealth remain limited.

To address this gap, we conducted a scoping review to provide an overview of the existing telehealth reimbursement policies across different countries, highlighting the various models and key considerations in designing these policies, such as the scope of coverage and payment standards. Additionally, with the advancement of internet technology, the global use of telehealth is steadily increasing, and many countries are actively promoting telehealth to enhance health care accessibility and strengthen their health care insurance systems. Introducing telehealth into health insurance reimbursement has become a significant trend. This scoping review offers insights into the major trends, challenges, and gaps in global telehealth reimbursement policies. By reviewing the current characteristics of telehealth insurance coverage, payment standards, and other relevant factors, this study provides a theoretical foundation for the development of telehealth reimbursement policies. It also serves as a valuable reference for best practices and policy recommendations.

## Methods

### Overview

We conducted this scoping review following the methodological framework proposed by Arksey and O’Malley [12], which comprises five stages: (1) identifying research questions; (2) relevant studies; (3) study selection; (4) data charting; and (5) collating, summarizing, and reporting the results. The reporting of the scoping review was guided by the PRISMA-ScR (Preferred Reporting Items for Systematic Reviews and Meta-Analyses extension for Scoping Reviews) checklist [13].

Narrative synthesis is a method for evaluating and synthesizing the results of multiple studies, primarily relying on the use of descriptive text to summarize and interpret research findings [14]. The application of narrative synthesis enables a clear presentation of the intricacies of telehealth reimbursement policies, thereby providing a robust foundation for the design of comprehensive reimbursement frameworks for telemedicine services.

### Search Strategy and Study Selection

We conducted a comprehensive search across China National Knowledge Infrastructure, Wan Fang, Weipu Database, Web of Science, PubMed, and Embase. Our search strategy used both Medical Subject Headings terms and free words from the inception of these databases until October 2023. The search algorithms (see Multimedia Appendix 1) were (telehealth OR telehealth OR Virtual medicine OR eHealth OR mHealth OR online diagnosis OR mobile health) AND (health insurance OR payment OR medicare OR reimbursement). References of the included studies were also reviewed to identify additional eligible studies.

The inclusion criteria for this scoping review included research related to telehealth research, involving topics such as telemedical services, internet hospitals, and diagnosis and treatment; research focused on the health insurance payment policies for telehealth services; research with clear health insurance payment methods including the scope, mechanisms, and entities involved in health insurance payments; and research with quantitative or qualitative methods. The exclusion criteria for this scoping review included nonresearch studies like news reprints and opinion pieces, inability to access the full text, research that only pertains to telehealth without specific mention of health insurance payment policies or practices, and research whose perspective is the combination of internet technology and health insurance payment.

The search process involved 3 stages—collection of studies, scanning of titles and abstracts, and reading of the full texts. Titles and abstracts retrieved from searches were independently screened by 2 authors. Full-text papers were also reviewed independently by the same 2 authors. Any disagreements were resolved through a joint meeting with a senior author to reach a consensus.

### Data Extraction and Charting the Data

The data were extracted from studies that met the eligibility and inclusion criteria for this review and recorded using a standardized Microsoft Excel data charting table. The data regarding the first author, date of publication, country, type of telehealth services introduced in health insurance, health insurance reimbursement providers, reimbursement standards for telehealth (eg, the condition for the reimbursement and reimbursement rate), and key findings of studies (see Multimedia Appendix 2) were extracted and analyzed.

### Collating, Summarizing, and Reporting the Results

We assessed the quality of each study using the PRISMA-ScR checklist (see Multimedia Appendix 3) and the Critical Appraisal Skills Programme (CASP) checklist (see Multimedia Appendix 4) [15]. Each criterion was scored as 1 (fully meeting the checklist standard) or 0 (not meeting or unclear about the checklist standard), resulting in a quality score for each study based on all criteria. Each scoping analysis received a total quality score of 8 points, and the components include title analysis, abstract, introduction, methods analysis, results analysis, and discussion analysis. Two researchers independently conducted the quality assessment of all papers, consulting a third researcher when necessary to reach a consensus. We used descriptive narrative synthesis combined with tabular summarization to present and interpret the research findings.

### Basic Definitions

The coverage area of health insurance payments refers to the geographical coverage of telehealth health insurance. The covered services under health insurance payment represent the types of services eligible for health insurance reimbursement. Standards of health insurance reimbursement for telehealth include the reimbursement rates and eligibility criteria for insurance coverage.

## Results

### Characteristics of the Studies

**Figure 1:**
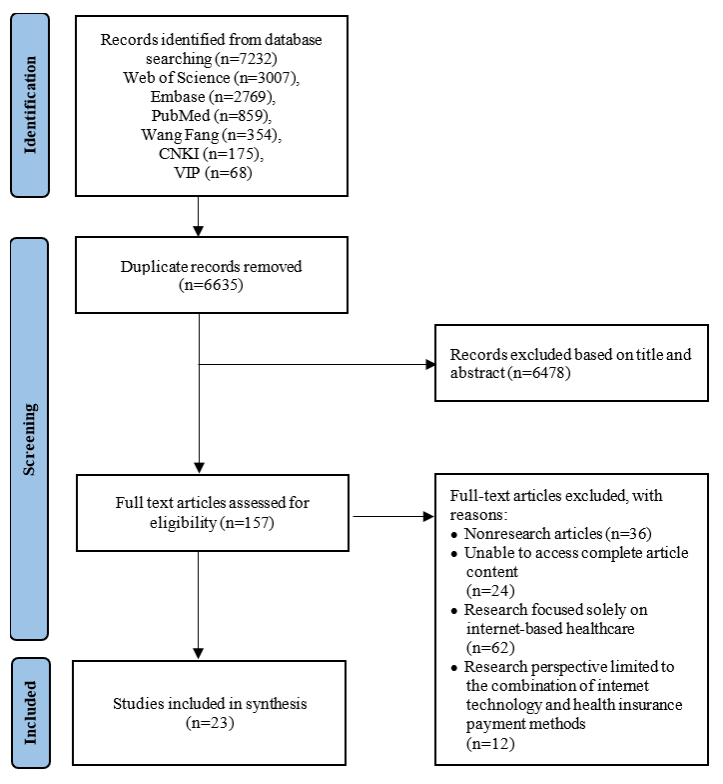
PRISMA (Preferred Reporting Items for Systematic reviews and Meta-Analyses) flowchart for the selection of the included studies. CNKI: China National Knowledge Infrastructure; VIP: Weipu Database.

A total of 7232 studies were initially identified in the database, consisting of 594 in Chinese and 6635 in English. Following the application of PRISMA-ScR criteria and deduplication using reference management software, a total of 5260 studies were retained (Figure 1). After strict adherence to inclusion and exclusion criteria, 23 studies were ultimately included [16-38], with 8 in Chinese and 15 in English. The selected studies spanned research conducted in the United States (n=8, 34%), China (n=6, 26%), Australia (n=3, 23%), France (n=3, 23%), Japan (n=2, 9%), Germany (n=2, 9%), and United Kingdom (n=1, 4%).

### Coverage Area of Health Insurance Payments

A total of 10 (43%) studies provided insights into the coverage of health insurance payments for telehealth across the examined regions. In China, the majority of provinces, cities, and districts have achieved fundamental coverage of telehealth under basic health insurance [16-19].

In the United States, the introduction of telehealth services in Medicare reimbursement categories dates back to the Balanced Budget Act of 1997. Initially, Medicare stipulated that the originating site (where patients receive telehealth services) must be located in health professional shortage areas or outside metropolitan statistical areas. However, in 2018, the Bipartisan Budget Act and the Patients and Communities Act lifted restrictions on the originating site, allowing it to be in any location. Currently, both Medicare and Medicaid reimburse telehealth services in all 50 states and the District of Columbia, with Medicaid allowing 14 states to consider homes as originating sites and 16 states allowing schools as originating sites. In 43 states and the District of Columbia, private payment laws for telehealth services have been enacted [4,20-22].

In Australia, telehealth services were introduced in the scope of health insurance reimbursement in 2011. Before the COVID-19 pandemic, only insured individuals residing in remote areas 2-5 could use telehealth services and receive reimbursement under the National Medicare Benefits Schedule (MBS). However, during the pandemic, reimbursement expanded to all insured individuals [23,24].

France introduced telehealth services in the scope of health insurance reimbursement in 2018. Insured individuals can be reimbursed for telehealth services just like regular health care. To ensure the treatment of telehealth services, patients who have undergone at least 1 in-person consultation within a year are permitted to use telehealth services. After the outbreak of the COVID-19 pandemic in 2020, all patients became eligible to access telehealth services [24,25].

In Germany, the Electronic Health Law in 2015 supported the adoption of telehealth, explicitly allowing reimbursement for telehealth services. In 2019, the Digital Healthcare Act (Digitale-Versorgung-Gesetz) granted all individuals covered by statutory health insurance the right to be reimbursed for eligible digital health applications, including video consultations, teleconsultations, remote monitoring, and remote secondary diagnoses. Digitale-Versorgung-Gesetz continues to promote the use of telehealth, benefiting all insured patients [26].

### Covered Services Under Health Insurance Payment

A total of 18 (78%) studies provided insights into the covered items under health insurance payments for telehealth services in the studied countries (as shown in Tables 1 and 2). In China, the definition of health insurance reimbursement scope varies across different regions based on local actions [16-18,27,28]. Covered services mainly included follow-up visits, remote consultations, remote monitoring, and remote diagnosis. Most regions introduced “electronic follow-up” services into health insurance payment, followed by “electronic consultation” services.

**Table 1:** Telehealth services covered by health insurance.

Region	Provider	Telehealth services
China	Basic Health Insurance	Telehealth follow-up services Remote consultation: single-discipline, multidisciplinary, synchronous, and asynchronous pathology consultation; traditional Chinese medicine syndrome differentiation; and treatment consultation Remote monitoring: fetal heart monitoring, remote ECG^a^ monitoring, pacemaker monitoring, and defibrillator monitoring Remote diagnosis: imaging diagnosis, pathology diagnosis, ECG diagnosis, and ultrasonography diagnosis
United States	Medicare, Medicaid, Private, Payer, and MA^b^ plans	Medicare: remote physiologic monitoring and treatment management, care management services (transitional care and chronic care), interprofessional health record consultations, psychological therapy, diagnosis and treatment of kidney diseases, medication assessment and management, and stroke telehealth Medicaid: store-and-forward (33 states), remote patient monitoring (34 states), audio-only calls (37 states and Washington, DC), live video (all 50 states and Washington, DC), and transmission or facility fee (35 states). Specific services limitations in some states Private payers: telehealth services reimbursed similarly to in-person services under parity state laws. Some states expanding commercial reimbursement policies MA plans: alternative to traditional Medicare, allowing private insurers an option to offer telehealth services under the PFS^c^ and following PFS usage rules. In addition, MA plans provide coverage for services via telehealth beyond what is required in original Medicare as additional telehealth benefits (eg, remote monitoring services for urban patients with multiple chronic diseases)
Australia	MBS^d^	2006-2019: sequential introduction of telehealth services into MBS. Video consultations for remote areas, mental health support for drought and bushfire-affected communities and advanced care for aged, cancer, and palliative patients COVID-19 epidemic: expansion of telehealth services in MBS. Early intervention, care for older people, pediatric, psychiatric, psychological, and dental diagnostic and treatment services
France	Assurance Maladie	Remote consultation: no professional restrictions on telehealth services Tele-expertise: a remote consultation among practicing physicians to remotely seek advice on patient treatment Remote monitoring: use of follow-up, particularly for remote monitoring of organ transplant patients
Japan	National health insurance	Remote consultation: among medical institutions and practicing physicians

^a^ECG: electrocardiogram.

^b^MA: Medicare Advantage.

^c^PFS: Physician Fee Schedule.

^d^MBS: Medicare Benefits Schedule.

**Table 2:** Regions covered by health insurance.

Region and telehealth services			Covered regions
**China**			
	Follow-up services	Beijing, Zhejiang, Jiangxi, Jilin, and 27 other provinces and municipalities	
	Remote consultation	Guangdong, Shanxi, Fujian, and 16 other provinces and municipalities	
	Remote monitoring	Sichuan, Tianjin, Shandong, and 16 other provinces and municipalities	
	Remote diagnosis	Jiangsu, Guizhou, Chongqing, and 7 other provinces and municipalities	
**United States**			
	Medicare	Nationwide	
	Medicaid	Nationwide	
	Private payers	40 states and the District of Columbia	
	Medicare advantage plans	Nationwide	
**Australia**			
	2006-2019	Nationwide	
	COVID-19 epidemic	Nationwide	
**France**			
	Remote consultation	Nationwide	
	Tele-expertise	Nationwide	
	Remote monitoring	Nationwide	
**Japan**			
	Remote consultation	Nationwide	

In the United States, health care insurance reimbursement is primarily through Medicare, Medicaid, private payer, and Medicare advantage plans, with specific payment items taking different forms between insurance types and regions. The Centers for Medicare and Medicaid Services (CMS) in the United States has established a list of Medicare telehealth services for reimbursement, which includes 268 permanent or temporary internet health care service items. These services typically cover consultations, remote monitoring, and mental health services. The list is updated annually and was expanded during the COVID-19 pandemic to include telehealth reimbursement items, such as remote cardiac and pulmonary rehabilitation therapies, enabling insured individuals to receive treatment services provided by clinicians from homes [17,20,29,30].

Australia primarily uses the MBS for health insurance payments, incorporating 11 specialized services (geriatrics, psychiatry, neurosurgery, obstetrics, gynecology, etc), 6 primary health care consultations, and 23 patient-end services. To address the COVID-19 pandemic, MBS gradually introduced internet health care service items and introduced approximately 300 MBS project numbers for remote health care consultations. Among them, 56 are for primary health care and 73 temporarily covered health care services during the pandemic have been permanently included in the health insurance plan [23,24,31].

In France, telehealth services are based on the scope of national health insurance, without specific subspecialty restrictions. During the pandemic, the insurance coverage for remote health care services was expanded. The Ministry of Health allows national health insurance (Assurance Maladie) to reimburse remote consultations and remote specialized knowledge for patients diagnosed with COVID-19 without the need for prior registration [24,25,32].

In the United Kingdom, the development of telehealth was relatively slow before the COVID-19 pandemic. After the outbreak, the surge in demand for telehealth prompted the National Health Service (NHS) to request supporting institutions such as health trusts and general practitioners to provide reimbursement support for telephone or digital video remote consultations [33].

Japan introduced remote health care services between medical service providers in health insurance payment, such as remote pathology and remote radiology diagnoses. For instance, in cases where a patient’s pathological tissue sample is received at the destination facility (affiliated hospital), and the examination requires the use of remote diagnostic technology from the sending facility (other medical institution), health insurance will cover the costs incurred by the sending facility for the rapid microscopic examination of the pathological tissue. Remote medical care for specific diseases is eligible for health insurance reimbursement, and some services of medical-patient remote medical care have gradually been introduced in health insurance payments after the COVID-19 pandemic [34].

### Standards of Health Insurance Reimbursement for Telehealth

A total of 10 (43%) studies provided insights into the standards of health insurance reimbursement for telehealth across various countries and health insurance payment standards for telehealth exhibit the following characteristics in different nations (as shown in Table 3).

The first characteristic is the establishment of health care insurance reimbursement quotas or ratios. In China, different provincial-level administrative regions have set varying insurance quotas or ratios. Some provinces and municipalities have set reimbursement amounts according to the level of hospitals or set a certain percentage of reimbursement according to the price of the service for health insurance [16,17,27]. In the United States, the CMS has established different payment systems for various entities, including the Physician Fee Schedule (PFS) and Fee-For-Service (FFS). Each system has distinct health insurance reimbursement conditions for telehealth medical services. The specific payment standards for each internet medical service are explicitly outlined in the PFS. Medicare relies on the payment standards of the PFS, compensating providers based on the unit of service delivered [4,20-22,30]. In France, national health insurance reimburses 70% of the costs for telehealth medical services, with individuals covering the remaining 30%. Certain populations, such as pregnant women and long-term care patients, receive full reimbursement from national insurance. During the COVID-19 pandemic period, the national health insurance fully covers diagnosed and suspected cases [24]. In Australia, the charging standard for telehealth services is set at 50% of the fee for in-person consultations. Physicians receive compensation through a bulk billing system, which means that patients incur no charges for receiving telehealth consultations. Physicians directly bill the MBS, and the MBS covers the full amount. Additionally, after the COVID-19 pandemic, there has been an expansion of full reimbursement for telehealth services, particularly benefiting the disadvantaged [24,31].

**Table 3:** The health insurance payment standards for telehealth services.

Region	Reimbursement quotas or ratios	Specific conditions
China	Reimbursement expenses per visit are claimed based on the hospital level. Beijing: follow-up services after reimbursement, ¥19 (approximately US $2.6) for primary hospital, ¥28 (approximately US $3.9) for secondary hospital, and ¥40 (approximately US $5.5) for tertiary hospital. Jiangxi: primary and tertiary hospitals can be reimbursed ¥13 (approximately US $1.8) and ¥9 (approximately US $1.2), respectively, for the follow-up services. According to the basic health insurance category A and category B list. Zhejiang: follow-up visits are in accordance with category A list. Henan: in accordance with category B list. Reimbursement is based on a certain percentage of the services price. Fujian: the reimbursement of consultations is set at 30% of the services price. Guizhou: the reimbursement of consultations is set at between 70% and 85% of services price for patients in relatively impoverished counties.	Reimbursement of specific services: Sichuan: reimbursement for remote ECGa and pacemaker monitoring is limited to patients with arrhythmia. Payment limits: Shaanxi and Guizhou: set annual payment limits. Fujian: a maximum of no more than ¥90 (approximately US $12.4) per visit. Visit duration limits: Shandong: reimbursement for remote monitoring service (excluding fetal heart monitoring) is limited to 24 hours per visit.
United States	The payment standards are listed in the physician fee schedule and are updated on an annual basis	Payment recipients: Medicare: aged 65 years and older, all patients with end-stage renal disease, and some individuals with disabilities. Medicaid: children, pregnant women, and the impoverished. Service locations: the originating site: within a Health Professional Shortage Area or a county outside of a metropolitan statistical area. In 2019, Centers for Medicare and Medicaid Services removed the geographic requirement for originating sites for patients with substance use disorder or co-occurring mental health.
Australia	Bulk-billing: service providers have the option to directly bill Medicare for their services without charging additional fees to patients.	Service locations: at least 15 km away from medical institutions
France	Under normal circumstances, the national health insurance covers 70% of the cost of telehealth services. Special populations (eg, pregnant women, long-term care patients, and covered by supplemental health insurance payment support plans) receive full coverage. During crises like the COVID-19 pandemic, the national health insurance covers 100% of the cost of telehealth services.	Patients who had in-person consultations within the past year services. Remote consultation equipment. Computer, tablet, or smartphone equipped with a webcam and connected to the internet, transmitting video data through a secure video platform. During the COVID-19 pandemic, allowance for the use of telephone for suspected patients with COVID-19, pregnant women, residents in “white zones” (areas without mobile phone or internet coverage), patients with chronic disease, and individuals aged older than 70 years.

^a^ECG: electrocardiogram.

The second characteristic is setting specific conditions. In some provinces and cities in China, restrictive conditions (eg, specific services and payment limits) for health insurance payment for telehealth services are designed to ensure the quality and safety of medical care. In the United States, there are conditions on populations and locations for health care [20,22,29]. France specifies that patients who have received in-person treatment or have been referred by primary care physicians can use reimbursed telehealth services. Patients must use communication devices with cameras and internet connectivity. However, conditions were relaxed after the COVID-19 pandemic, eliminating the mandatory requirement for patients to have in-person initial consultations and allowing certain populations to consult via telephone to address inequalities in digital access [24,31]. In Australia, telehealth services are not accepted for hospitalized patients. Moreover, eligibility for health insurance reimbursement is limited to patients located at least 15 kilometers away from medical institutions who receive such services [23,24].

### Differences in Health Insurance Payments for Telehealth Services and In-Person Services

A total of 4 (17%) studies reported the differences in health insurance payment between telehealth and in-person health care services in various countries. First, some regions in China such as Sichuan and Shandong provinces, adopt the same health insurance payment standard for telehealth as for in-person services [27]. Similarly, countries like France, Luxembourg, the Netherlands, and Switzerland maintain equivalent payment standards for telehealth consultations compared to in-person services [32]. Additionally, states in the United States, including Tennessee and Michigan, have enacted laws establishing parity for telehealth, guiding reimbursement for telehealth consultations [36]. When formulating parity policies, factors such as resource use and costs related to telehealth services, demand for alternative payment models, and differences in outcomes between telehealth care and in-person care are typically taken into consideration [37]. Second, telehealth services often adopt reimbursement levels lower than those for in-person services [35]. A survey of telepsychiatry consultations provided in 17 countries and territories found that the degree of telepsychiatry reimbursement rates from public health insurance was lower than in-person service in many countries, such as Japan, even after the outbreak of the COVID-19 pandemic [38].

## Discussion

### Principal Findings

The review identified the current status of health insurance payments for telehealth services. Our study found that an increasing number of countries are incorporating telehealth into their health insurance systems. However, there are significant differences in reimbursement standards and conditions across different countries.

The COVID-19 pandemic has significantly accelerated the global adoption of telehealth, profoundly impacting health care service models. Many countries have expanded their health insurance policies to include telehealth, recognizing its importance in maintaining the continuity of care during the pandemic. As detailed in the results, several key changes occurred during the pandemic [10,11,22,24,33,34]. The pandemic prompted many health insurance systems to expand their coverage to include a broader range of telehealth services, thereby addressing the increased demand for remote consultations. Additionally, geographical restrictions were often relaxed, allowing patients in more regions to access reimbursed telehealth services.

Moreover, the reimbursement rates for these services were generally increased to ensure they were on par with the in-person consultations. These adjustments were crucial in maintaining access to essential health care services while minimizing the risk of COVID-19 transmission. Overall, the pandemic has accelerated the integration of telehealth into mainstream health care delivery, highlighting the need for adaptable health insurance policies capable of responding to PHE.

The study found that some countries, such as the United States and Australia, have more detailed distinctions in reimbursement items, considering further stratification for specialized services. This is somewhat related to the diagnosis-related groups (DRG) health insurance payment policies implemented by these countries. Such reimbursement strategies help achieve standardization of medical services and rational use of medical resources, ensuring that patients can receive appropriate medical care. However, there are few researches on the design of health care insurance reimbursement frameworks for telehealth services. Many countries lack unified standard regulations, which grants local governments significant flexibility to integrate telehealth services into the existing health insurance systems. Determining reasonable payment standards has always been a topic of keen interest across various sectors of society.

We found that some regions adopt reimbursement methods identical to in-person services, integrating telehealth insurance reimbursement into the overall in-person health care insurance budget for unified management [39]. This approach can promote the development of telehealth and better integrate telehealth services with in-person medical services.

The results also revealed that telehealth services included in health insurance have gradually diversified and the number of disciplines covered has increased, which is a reflection of the digital transformation of health care services and the adaptive adjustment of health insurance policies. It is worth noting that some regions are incorporating telehealth follow-up services for chronic diseases into health insurance reimbursement [27,30]. Encouraging patients with chronic diseases to choose telehealth follow-up appointments can save their time, as well as reduce the wastage of hospital resources. Receiving services is the first part of health insurance payment, but there still leaves many services unaddressed such as some prescription drugs and maternity care, which are not covered by health insurance reimbursement.

Some studies have emphasized the barriers faced in the actual implementation of health insurance reimbursement for telehealth. First, it faces risks to medical quality, safety, and information security, necessitating further protection of patient rights. Moreover, in the open environment of the internet, there is a risk of third-party theft and leakage of patient consultation information, health insurance records, and other personal privacy. Second, in the absence of targeted supervision and information asymmetry, it may lead to excessive health care consumption and fraudulent claims for health insurance reimbursement, which can result in unreasonable growth in medical expenses, leading to waste of health insurance funds and inefficiency in the allocation of medical resources [40].

### Comparisons to Existing Literature

Our results are consistent with existing findings. Policy makers often introduce additional flexibility to facilitate patient reimbursement for telehealth services during the PHE. These adjustments were widely adopted during the pandemic, leading to the rapid expansion of telehealth, which has now become an integral part of routine health care in many regions. The lessons learned from these flexible policies have prompted policy makers in various countries to reevaluate telehealth reimbursement strategies, with considerations to maintain or further enhance these measures in the post-pandemic era to support the long-term growth of telehealth [41]. The differing definitions of telehealth may lead to inconsistencies in subsequent regulations, guidelines, and reimbursement policies [42]. As our study shows, the variation in telehealth definitions across regions results in differences in the services covered by insurance and the associated payment policies. These disparities could lead to significant differences in the development and adoption of telehealth services across regions, presenting challenges for the implementation of telehealth services across regional and national borders.

For payers of health insurance, a key concern is whether telehealth will increase expenditures [43]. The impact of telemedicine on costs is also influenced by the differences in expenses between telehealth and in-person services. Our research shows that most countries or regions have aligned the reimbursement levels for telehealth with those for equivalent in-person visits. The level of reimbursement for telehealth depends on factors such as the patient population, health care environment, specific conditions, and modes of interaction. The ultimate goal of health insurance payment policies is to integrate telehealth and in-person services effectively, aiming to balance and harmonize these 2 service models to enhance accessibility to medical services while managing the balance of insurance revenues and expenditures.

### Policy Implications

Our study had several policy implications. First, it is necessary to consider various factors such as medical demand, fairness, efficiency, and the financial capacity of the health insurance fund to establish scientific and feasible admission standards and formulate a unified and reasonable catalog of telehealth services covered by health insurance. Second, a well-implemented health insurance payment mechanism is crucial for promoting the widespread adoption of telehealth services with a lower economic burden on patients. Telehealth, as an emerging form of health care service, relies on the national health insurance policy framework for its insurance policy formulation. Existing payment methods include FFS, capitation, global budget payment, DRG, and diagnosis-intervention packet. Each payment method has its advantages and disadvantages in different countries and health care systems. FFS can encourage doctors to provide medical services and take on risks [44] but may lead to inefficient over-provision or under-provision of some medical services, as well as rapid increases in health care costs [45]. Capitation and global budgets can effectively control the growth of health care costs and improve efficiency but may impact the quality of medical services [44]. DRG and diagnosis-intervention packets help control health care costs and improve service quality but can inevitably lead to issues such as upcoding and cherry-picking patients [46,47]. Different countries are at varying stages of telehealth development, and the suitable health insurance payment methods differ accordingly. In the initial stage, where telehealth is in its infancy, the technical foundation is weak, regulations are underdeveloped, and public awareness and acceptance are low. At this stage, FFS payments, prepaid card systems, and single payment channels are recommended as they are easy to manage and understand. As the technical foundation of the internet improves, telehealth gradually becomes more widespread. The number of users increases, and overall health care costs rise, placing higher demands on the management and use of health insurance funds. Therefore, we recommend implementing capitation, global budget payment methods, and DRG, during this stage, as these can effectively control the rapid growth of health care expenses. In the advanced stage, where internet technology is mature and telehealth is highly developed, the goal is to reduce the growth rate of health care costs while improving medical quality and health outcomes. Therefore, we suggest adopting pay-for-performance methods and linking payment amounts to medical quality and performance to establish a more comprehensive and integrated payment system. Currently, many countries are exploring value-based payment methods to optimize existing health insurance payment models. Overall, we think that the enhancement of telehealth technology is crucial for the improvement of the health insurance payment system. Furthermore, policy makers should integrate the above payment methods according to the characteristics of different telehealth services and consider adopting mixed payment models to optimize deductibles, maximum limits, and copayment ratios in telehealth insurance payments to achieve better results. Third, enhancing the connectivity between telehealth and in-person health insurance payments, and reducing the disparity in reimbursement ratios between telehealth and in-person services to encourage the development of telehealth services are essential. Finally, we recommend promoting the construction of intelligent supervision systems for health insurance, establishing monitoring rules and indicators, and using big data to conduct real-time supervision and control of diagnosis and treatment behaviors, which will ensure the secure use of health insurance funds.

### Limitations

Due to limitations in resources and data availability, this review did not include the health care insurance systems for some countries. The inclusion of studies was limited to those available in English or Chinese, which may have led to the omission of some relevant studies published in other languages. Finally, the review only conducted a qualitative analysis of the included studies, and the quality of the methods reported may introduce bias into the research findings.

### Conclusions

The variability in reimbursement practices we observed highlights the need for more standardized guidelines to support the development of telehealth services. We recommend that future scoping reviews broaden the scope and perspective of research to include a wider range of countries, providing a more comprehensive understanding of global telehealth insurance systems. This approach would offer more precise insights for improving telehealth insurance policies, particularly in countries and regions with varying income levels.

## Appendix

Multimedia Appendix 1 Search strategy.

Multimedia Appendix 2 Characteristic of the included studies and data extraction.

Multimedia Appendix 3 PRISM-ScR (Preferred Reporting Items for Systematic reviews and Meta-Analyses extension for Scoping Reviews) checklist.

Multimedia Appendix 4 Critical Appraisal Skills Programme (CASP) checklist.

## Abbreviations

CASP: Critical Appraisal Skills Programme
CMS: Centers for Medicare and Medicaid Services
DRG: diagnosis-related group
FFS: Fee-For-Service
MBS: Medicare Benefits Schedule
NHS: National Health Service
PFS: Physician Fee Schedule
PHE: public health emergencies
PRISMA-ScR: Preferred Reporting Items for Systematic Reviews and Meta-Analyses extension for Scoping Reviews
WHO: World Health Organization
