# Supplementary material for: Health Insurance Payment for Telehealth Services: Scoping Review and Narrative Synthesis
Source: J Med Internet Res. 2024 Dec 9;26:e56699. doi: 10.2196/56699 (PMC11668521; doi:10.2196/56699)
Supplement: Multimedia Appendix 1 [file jmir_v26i1e56699_app1.docx]

| **Datebase name** | **Literature search timeframe /**  **Date of search** | **Search algorithms** |
| --- | --- | --- |
| Web of Science | From inception of database to 31 October 2023/  October 2023 | (TS=((Telemedicine) OR (Virtual Medicine) OR (telehealth) OR (mHealth) OR (eHealth) OR (online diagnosis) OR (mobile health))) AND TS=((medical insurance) OR (payment) OR (medicare)OR (reimbursement)) |
| Pubmed | From inception of database to 31 October 2023/  October 2023 | (((((((Telemedicine[MeSH Terms]) OR (Virtual Medicine[Title/Abstract])) OR (telehealth[Title/Abstract])) OR (mHealth[Title/Abstract])) OR (eHealth[Title/Abstract])) OR (online diagnosis[Title/Abstract])) OR (mobile health[Title/Abstract])) AND (((medical insurance[Title/Abstract]) OR (payment[Title/Abstract])) OR (medicare[Title/Abstract])) |
| Embase | From inception of database to 31 October 2023/  October 2023 | #1: telemedicine OR (virtual AND medicine) OR telehealth OR mhealth OR ehealth OR (online AND diagnosis) OR (mobile health)  #2: medical insurance OR payment OR medicare OR reimbursement  #3: #1 AND #2 |
| CNKI | From inception of database to 31 October 2023/  October 2023 | （主题：互联网医疗 + 远程医疗 + 虚拟医疗 + 互联网医院）AND（主题：医保支付 + 医保移动支付 + 医保结算） |
| WAN FANG | From inception of database to 31 October 2023/  October 2023 | （互联网医疗 OR 远程医疗 OR 虚拟医疗 OR互联网医院）AND（医保支付 OR 医保移动支付 OR 医保结算） |
| VIP | From inception of database to 31 October 2023/  October 2023 | （互联网医疗 OR 远程医疗 OR 虚拟医疗 OR互联网医院）AND（医保支付 OR 医保移动支付 OR 医保结算） |
