# Supplementary material for: Health Insurance Payment for Telehealth Services: Scoping Review and Narrative Synthesis
Source: J Med Internet Res. 2024 Dec 9;26:e56699. doi: 10.2196/56699 (PMC11668521; doi:10.2196/56699)
Supplement: Multimedia Appendix 2 [file jmir_v26i1e56699_app2.docx]

| Author/Year | Country | Language | Study method | Aim | Covered telehealth services | Reimbursement standards for telehealth services | Health insurance reimbursement Providers | Key findings |
| --- | --- | --- | --- | --- | --- | --- | --- | --- |
| Li GY, Yao Q, Liu XJ, et al. (2023)  [16] | China | Chinese | Content analysis and cluster analysis methods | Analyze the current state of medical insurance payment policies for internet-based healthcare services across different provinces in China | Remote fetal monitoring, remote ECG monitoring, remote pacemaker monitoring, remote defibrillator monitoring, home automatic peritoneal dialysis monitoring,et al | Reimbursement standards vary widely among provinces. For example, Sichuan reimburses remote ECG and pacemaker monitoring only for arrhythmia patients. | Basic Health Insurance | Significant differences in insurance coverage across provinces. Great all coverage but with many restrictions. |
| Ding TT, Qian AB, Tan ZX (2022)  [17] | China | Chinese | Comparative analysis of domestic and international policies | Examine the issues in the reimbursement of telehealth services by health insurance. | Internet-based consultations, online follow-up consultations for chronic diseases, remote consultations,et al | China: Varies by region and type of service.  United States: The Centers for Medicare and Medicaid Services (CMS) provides a clear list of telehealth services eligible for reimbursement, updated annually. The reimbursement is standardized using a formula that considers regional cost adjustments. | Basic Health Insurance, Medicare | There is considerable variation in pricing and reimbursement standards across different regions in China. Some regions have well-defined standards, while others are still in the exploratory phase. The study suggests clarifying service items and pricing mechanisms, improving reimbursement scopes and models, optimizing chronic disease insurance for the elderly, enhancing external support systems, and strengthening regulatory capacities. |
| Liang HM, Xu JJ, Peng Y, et al. (2020)  [18] | China | Chinese | Comparative analysis of the practices and experiences of China and the United States | Analyze the evolution and current status of internet medical service pricing policies, explore issues in reimbursement for internet medical services | Follow-up consultations, remote consultations, remote pathology consultations, and remote monitoring | The reimbursement standards for telehealth services vary by region. For instance, Shandong charges remote monitoring by the hour, while Sichuan charges per session, with fees differing based on the medical institution's level and the doctor's rank. Guidelines from the National Healthcare Security Administration and National Health Commission state that eligible telehealth services can be covered by medical insurance. | Basic Health Insurance, National Healthcare Security Administration and local healthcare insurance departments | China: There is a lag in the formulation and implementation of pricing and reimbursement policies for internet medical services, and there is an urgent need for standardized and unified price standards and reimbursement models​​.  United States: The scope of reimbursement for telehealth services has been gradually expanded, and policy adjustments have promoted the rapid development of telehealth​ |
| Cui WB, Zhang KK, Gu ST, et al. (2020)  [19] | China | Chinese | Policy analysis | Provide policy suggestions for incorporating "Internet Plus" medical services into medical insurance payments. | Remote consultations, remote treatments, and rehabilitation services,et al | The reimbursement standards for telehealth services involve the development of price setting mechanisms for different types of telehealth services. | Public and private medical institutions | The key findings highlight the significant role of "Internet Plus" medical services in improving healthcare accessibility, especially during the COVID-19 pandemic. The study notes the rapid development of these services and their inclusion in health insurance reimbursement systems in various provinces. It also points out the challenges faced, such as pricing, payment methods, and risk management, and provides detailed policy suggestions to address these issues. |
| Liu Y, Li Y, Jiang X, et al. (2021)  [20] | USA | Chinese | Comprehensive policy analysis | Analyze the U.S. telehealth services' insurance reimbursement policies and draw lessons to enhance the Chinese medical insurance system's integration with telehealth services | Real-time video services, Store-and-forward services, Remote patient monitoring,et al | In the U.S., telehealth reimbursement standards vary by insurance type | Medicare and Medicaid, Private Insurers | Policy Evolution: U.S. telehealth policies have progressively expanded since the 1990s, with significant milestones like the Balanced Budget Act of 1997 and the Affordable Care Act of 2010, which broadened the scope and reimbursement levels for telehealth services.  State Variations: There are significant differences in telehealth reimbursement policies across states, with some states offering comprehensive coverage and others having more restrictive policies.  COVID-19 Impact: The COVID-19 pandemic accelerated the relaxation of regulatory barriers, expanding telehealth service coverage and reimbursement flexibility across the country. |
| Park S, Langellier BA, Burke RE (2021)  [21] | USA | English | Qualitative study using cross-sectional data from four CMS data sources for 2020 | Analyze new telehealth benefits offered by Medicare Advantage (MA) plans in 2020 and examine plan characteristics associated with the provision of these new telehealth benefits. | Primary care services, mental health services, and urgent care services,et al | Overall, 86% of MA plans required a copay for telehealth services with an average copay of $12.84. Another 6% required a coinsurance payment, with an average coinsurance rate of 19.69%. | MA plans | Of 2992 unique MA plans, 58.1% offered new telehealth benefits in 2020. The new telehealth benefits were not more commonly offered by plans in rural areas |
| Abbasi-Feinberg F (2020)  [22] | USA | English | Comprehensive policy analysis | Explore the current and future trends in telemedicine coding and reimbursement | Online digital evaluation, remote physiologic monitoring and treatment management services,et al | Reimbursement standards for telehealth services vary by payer and state. Medicare primarily reimburses for real-time interactive encounters, while Medicaid and commercial payers may allow for more flexibility. | Medicare,Medicare Advantage, Medicaid, Commercial Payers, Employer-Paid Plans, Self-Pay Models | Telehealth coding and reimbursement rules vary by payer and state, and providers need to continuously monitor this rapidly changing landscape. During the COVID-19 pandemic, CMS and many private payers relaxed restrictions on telehealth services, which may have long-term implications on future policies. |
| Fisher K, Davey AR, Magin P (2022)  [23] | Australia | English | Narrative review | Examine the use of telehealth in general practice in Australia and explore the benefits, barriers, and facilitators of performing telehealth consultations. | Videoconferencing and telephone consultations, remote patient monitoring,et al | Reimbursement standards for telehealth services were significantly influenced by the introduction of Medicare Benefits Schedule (MBS) item numbers. Initially, 281 temporary MBS telehealth item numbers were introduced in response to the COVID-19 pandemic, of which 56 were for primary care. | Medicare Benefits Schedule (MBS) | Telehealth increased access to healthcare, particularly for vulnerable populations, and reduced the risk of COVID-19 transmission.  Facilitators for successful telehealth implementation included tailored GP training and sustainable funding models. |
| Suk K jin, Mi LS (2022)  [24] | France, Australia | Korean | Comparative analysis research method | Examine the telehealth policies of France and Australia before and after the COVID-19 pandemic | A variety of medical consultations and treatments conducted remotely using information and communication technology. | France: Telehealth services are reimbursed by the national health insurance, covering 70% of the consultation fees, with specific conditions for full coverage in certain cases like pregnancy, long-term care, and universal health insurance beneficiaries. During the COVID-19 pandemic, the government covered 100% of the telehealth consultation fees. | The national health insurance, Medicare | The COVID-19 pandemic led to the relaxation of previous restrictions and the expansion of telehealth services in both countries. France and Australia both had legal frameworks in place for telemedicine before the pandemic, but these were significantly adapted to meet the increased demand and changing healthcare needs during the pandemic. |
| Seon J kwan (2022)  [25] | France | Korean | Qualitative analysis | Provide insights into the telemedicine legislative framework in France and to derive implications for the potential revision of telemedicine laws in South Korea. | Teleconsultation, Telemonitoring,Teleassistance, Telesurveillance | Reimbursement standards for telehealth services in France are outlined in several legislative documents and decrees. These standards ensure that telehealth services are reimbursed similarly to in-person consultations, provided they meet specific criteria established by the national health insurance system. The study highlights that the French government has progressively expanded reimbursement policies to include a broader range of telehealth services, particularly in response to the COVID-19 pandemic. | The national health insurance system (L'Assurance Maladie) | Policy Adaptations: The COVID-19 pandemic prompted significant adaptations in telehealth policies, leading to expanded coverage and reimbursement for telehealth services.  Implementation Challenges: Despite the legislative support, challenges remain in the implementation of telehealth services, including issues related to technology infrastructure, patient and provider acceptance, and regulatory compliance. |
| Gerke S, Stern AD, Minssen T (2020)  [26] | Germany | English | Comprehensive policy analysis | Examine the changes brought about by Germany's Digital Healthcare Act (DVG) in the context of the COVID-19 pandemic, and to explore the implications and opportunities these changes present for other countries. | Teleconsultations, Remote monitoring,et al | The reimbursement process involves initial use of the manufacturer's list price for the first year, followed by a negotiated price that applies to all statutory health insurers. | The primary providers for health insurance reimbursement in Germany are the statutory health insurance funds. Approximately 90% of the population is covered by these state-funded insurers. | COVID-19 Impact: The pandemic accelerated the adoption and expansion of telehealth services, with temporary measures allowing greater flexibility in telehealth consultations and coverage.  Privacy Concerns: Issues related to data privacy and security are critical, with specific requirements for data protection and state-of-the-art security measures. |
| Cui WB, Zhang KK, Gu ST, et al. (2020)  [27] | China | Chinese | Literature review and policy analysis | Investigate how to incorporate "Internet +" medical services into the medical insurance reimbursement system to promote sustainable development of internet healthcare services. | Remote consultations and diagnoses, follow-up services for common and chronic diseases. | The study found variations in reimbursement policies across different regions. | Basic Health Insurance | Reimbursement Inclusion: Guizhou and Ningxia have included internet medical services in their medical insurance reimbursement systems, while Sichuan and Jiangsu are in the process of exploring similar inclusions.  Pricing Standards: The study emphasizes the need for reasonable pricing standards to ensure sustainable development, suggesting a combination of government and market-based pricing mechanisms.  Quality and Safety: Effective supervision and regulatory measures are necessary to ensure the quality and safety of "Internet +" medical services and the security of medical insurance funds. |
| Ma SQ, Cao Y (2021)  [28] | China | Chinese | Literature review and policy analysis | Investigate the current status of "Internet +" medical services in China, identify the challenges in their integration into the medical insurance system, and propose policy recommendations to facilitate their development and reimbursement | Remote consultations and diagnoses, follow-up services,et al | Reimbursement standards for telehealth services vary by region. | Basic Health Insurance | Reimbursement Inclusion: Provinces like Guizhou and Ningxia have included internet medical services in their medical insurance reimbursement systems, while others like Sichuan and Jiangsu are exploring similar inclusions.  Pricing Standards: The study emphasizes the need for reasonable pricing standards to ensure sustainable development, suggesting a combination of government and market-based pricing mechanisms.  Quality and Safety: Effective supervision and regulatory measures are necessary to ensure the quality and safety of "Internet +" medical services and the security of medical insurance funds. |
| Xiao GJ, Xu HF (2022)  [29] | USA | Chinese | Comprehensive analysis | By analyzing the policies and practices of Medicare in the United States, the study seeks to offer strategic guidance on enhancing the development and reimbursement of internet medical services in China. | Synchronous Telehealth Services, Remote Patient Monitoring, Asynchronous Telehealth Services,et al | Geographical Restrictions: Initially limited to rural areas but expanded during the COVID-19 pandemic.  Service Coverage: Limited to specific services listed in the Physician Fee Schedule (PFS).  Payment Models: Includes fee-for-service (FFS), bundled payments, and value-based payments under different Medicare parts (Part A, Part B, and Medicare Advantage plans)​. | Medicare | Policy Evolution: Medicare has progressively expanded telehealth service coverage, especially during the COVID-19 pandemic.  Reimbursement Flexibility: Medicare Advantage plans have greater flexibility in offering additional telehealth services compared to traditional Medicare. |
| Bogulski CA, Acharya M, Pro G (2023)  [30] | USA | English | A cross-sectional design to analyze disparities in telehealth utilization among Medicare beneficiaries in Arkansas in 2019. | Identify and understand the disparities in telehealth utilization among Medicare beneficiaries in Arkansas, focusing on how race/ethnicity, rurality, and chronic conditions intersect to influence access and usage of telehealth services | Primary care, specialist consultations, mental health services,et al | Medicare reimbursement standards for telehealth services typically require the use of real-time interactive audio and video communications. The study noted that during the pandemic, Medicare expanded the range of reimbursable telehealth services and relaxed some requirements, which significantly influenced utilization rates. | Medicare | Disparities in Utilization: The study found significant disparities in telehealth utilization based on race/ethnicity, with Black and Hispanic beneficiaries being less likely to use telehealth services compared to White beneficiaries.  Impact of Rurality: Beneficiaries living in rural areas had lower telehealth utilization rates than those in urban areas, highlighting geographic barriers to accessing telehealth services.  Chronic Conditions: Individuals with multiple chronic conditions were more likely to use telehealth services, indicating that those with greater healthcare needs were more inclined to utilize telehealth. |
| Hall Dykgraaf S, Desborough J, de Toca L (2021)  [31] | Australia | English | Narrative review | Review the literature on telehealth implementation in Australia, outline the pre-COVID background of telehealth services, and describe the national telehealth policy measures instituted in response to the COVID-19 pandemic. | Telephone and video consultations, Mental health support,etal | Reimbursement standards for telehealth services during the COVID-19 pandemic included:Introduction of approximately 300 temporary Medicare-subsidized telehealth services.Services were initially bulk-billed, meaning no out-of-pocket costs for patients, with incentives provided to support healthcare providers. The reimbursement policy evolved to allow private billing alongside bulk-billing, to ensure the financial viability of medical practices. | MBS | In Australia during the COVID-19 pandemic, telehealth consulting has been supported by a national policy response focused on payment for services through the nation’s universal health insurance scheme. |
| Raes S, Trybou J, Annemans L (2022)  [32] | Multiple | English | Comprehensive policy analysis | Examine telemedicine payment systems across ten health systems | Telemonitoring, Tele-expertise,et al | Reimbursement standards for telehealth services varied across the analyzed countries. | National health insurance | Many countries did not provide payment for telemonitoring and tele-expertise, missing the potential benefits of these technologies.Few countries established payment parity between televisits and in-person visits. Lack of parity could discourage the use of televisits due to lower reimbursement rates.The dominant payment system for telemedicine was fee-for-service (FFS), which could lead to overconsumption and other challenges. Capitation-based or hybrid systems, though less common, were suggested as alternatives to address these challenges. |
| Fisk M, Livingstone A, Pit SW (2020)  [33] | Australia, UK, USA | English | Comprehensive policy analysis | Examine how Australia, the United Kingdom, and the United States promoted telehealth during the early stages of the COVID-19 pandemic to support the identification and treatment of COVID-19 | Health screening, mental health counseling, remote patient monitoring,et al | The new Medicare rules in the United States required telehealth services to consult in real-time, removing the prior restriction to rural and remote areas. Reimbursement rates for telehealth services were set at the same rate as face-to-face visits. Private insurance providers also started waiving costs for remote assessments and consultations​​. | National health insurance | Key findings highlighted the rapid adoption and scaling of telehealth services in response to COVID-19. There were notable differences in how telehealth was integrated into health systems across the three countries. The study also noted the potential long-term integration of telehealth into healthcare frameworks due to the pandemic, despite existing barriers such as technology access, security concerns, and the need for suitable business models and funding. |
| Kim JS, Oh SH (2018)  [34] | USA, Japan, Korea | Korean | Comparative analysis | The aim of the study is to derive policy implications for Korea by comparing and analyzing the telemedicine policies and implementation experiences of the United States and Japan, which are ahead in conducting telehealth. | Teleconsultation, remote patient monitoring, telepsychiatry, and telepharmacy. | United States: Reimbursements are detailed for various services under Medicare and Medicaid. For instance, specific HCPCS and CPT codes are assigned for teleconsultations and other telehealth services.  Japan: Telehealth services are reimbursed when they supplement face-to-face medical services. Detailed guidelines specify the conditions under which telehealth services are eligible for reimbursement.  Korea: The policies are less developed, and reimbursement standards are not as clearly defined compared to the United States and Japan. | Medicare and Medicaid, National health insurance | The United States and Japan have established detailed and rigorous telehealth policies covering legal, financial, and operational aspects. |
| Wilson FA, Rampa S, Trout KE(2017)  [35] | USA | English | Analysis of private claims data from the Health Care Cost Institute (HCCI) for telehealth vs. non-telehealth services from 2009 to 2013. | To compare reimbursements between telehealth and non-telehealth services in the United States. | Evaluation and management (E/M), psychiatric diagnostic interviewing, psychotherapy. | Telehealth reimbursements for office visits for evaluation and management of established patients with low complexity were 30% lower than the corresponding non-telehealth service. | Private Payers | Telehealth reimbursements are significantly lower than non-telehealth for most common services; widespread telehealth adoption may be hindered by lower reimbursements. |
| Neufeld JD, Doarn CR, Aly R (2016)  [36] | USA | English | A mixed-method approach combining qualitative and quantitative data collection techniques | Evaluate the effectiveness of telehealth services and alternative payment models in improving healthcare delivery | Emote patient monitoring, virtual consultations, and tele-ICU services | Reimbursement standards varied by state and payer but generally included fee-for-service, bundled payments, and value-based reimbursement models. | Medicare, Medicaid, and various private insurers | Although Medicare telemedicine policy has changed little since its inception, changes in state policies with regard to telemedicine reimbursement appear to have significant impacts on the practical viability of telemedicine programs that bill Medicare for telemedicine services. |
| Khera N, Knoedler M, Meier SK (2023)  [37] | USA | English | Qualitative analysis | The aim of the article is to address the challenges and necessary steps to achieve payment and coverage parity for virtual care and in-person care. | Video visits, audio-only telephone services, remote patient monitoring, and other digital health interventions | Reimbursement standards for telehealth services vary significantly across states. Some states have enacted payment parity laws requiring insurers to reimburse telehealth services at the same rate as in-person services, while others have not. | Medicare, Medicaid and private insurers | There is significant variability in how telehealth services are covered and reimbursed across different states and insurers. This inconsistency can discourage providers from offering telehealth services.The COVID-19 PHE led to a surge in telehealth adoption due to relaxed restrictions and expanded coverage. |
| Kinoshita S, Cortright K, Crawford A (2022)  [38] | Multiple | English | Quantitative study | Clarify the telepsychiatry regulations for each participating country/region before and during the COVID-19 pandemic to understand how these changes impacted the implementation and use of telepsychiatry globally | Telepsychiatry | Initially, lower insurance reimbursement for telepsychiatry compared to in-person consultations was common in many regions. During the pandemic, 15 of the 17 regions reported that telepsychiatry services were reimbursed at the same rate (or higher) as in-person consultations. Some regions, like India, South Korea, and Spain, which did not previously provide insurance reimbursement for telepsychiatry, began doing so during the pandemic. | National health insurance | Telepsychiatry services were reimbursed at the same rate (or higher) as in-person consultations in 15 regions during the pandemic. Many regions relaxed restrictions on patient eligibility for telepsychiatry, making it more accessible. |

**Reference**

16. Li GY, Yao Q, Liu XJ, et al. Research on Medical Insurance Payment Policy of "Internet Plus" Medical Service in China [J]. Health Economics Research, 2023, 40(05): 43-46. https://d.wanfangdata.com.cn/periodical/wsjjyj202305011

17. Ding TT, Qian AB, Tan ZX. Comparative analysis of domestic and foreign Internet medical service pricing and medical insurance payment policies[J]. Chinese Hospitals, 2022, 26(9): 10-13. Doi:10.19660/j.issn.1671-0592.2022.9.03 https://d.wanfangdata.com.cn/periodical/ChlQZXJpb2RpY2FsQ0hJTmV3UzIwMjQwNzA0EhF6aG9uZ2d5eTIwMjIwOTAwMxoIN3hnM2RsdXY%3D

18. Liang HM, Xu JJ, Peng Y, et al. Analysis on the Core Issues of Pricing and Payment of "Internet+” Medical Services in China [J]. Chinese Health Economics, 2020(12), 39: 52-56. DOI: 10.7664/CHE20201212 https://d.wanfangdata.com.cn/periodical/ChlQZXJpb2RpY2FsQ0hJTmV3UzIwMjQwNzA0Eg96Z3dzamoyMDIwMTIwMTQaCGJhaGVsYWdz

19. Cui WB, Zhang KK, Gu ST, et al. Policy suggestions on inclusion of "internet +” medical services into medical insurance payment [J]. Chinese Health Resources, 2020, 23(2): 102-105+147. DOI: 10.3969/j.issn.1007-953X.2020.02.004 https://d.wanfangdata.com.cn/periodical/ChlQZXJpb2RpY2FsQ0hJTmV3UzIwMjQwNzA0Eg96Z3dzenkyMDIwMDIwMDQaCDc2a2FjaWE3

20. Liu Y, Li Y, Jiang X, et al. Analysis and enlightenment of health insurance payment policy for Internet medical services in the United States[J]. Chinese Journal of Hospital Administration, 2021, 37(8): 642-646. DOI: 10.3760/cma.j.cn111325-20210303-00173 https://d.wanfangdata.com.cn/periodical/ChlQZXJpb2RpY2FsQ0hJTmV3UzIwMjQwNzA0Eg96aHl5Z2wyMDIxMDgwMDYaCHQ2M2Ezdnly

21. Park S, Langellier B A, Burke R E. Telehealth Benefits Offered by Medicare Advantage Plans in 2020[J]. Medical Care, 2021, 59(1): 53-57. DOI:10.1097/MLR.00000000000001408

22. Abbasi-Feinberg F. Telemedicine Coding and Reimbursement - Current and Future Trends [J]. Sleep Medicine Clinics, 2020, 15(3): 417-429. PMID:32762974

23. Fisher K, Davey A R, Magin P. Telehealth for Australian general practice: The present and the future[J]. Australian Journal of General Practice, 2022, 51(8): 626-629. DOI: 10.31128/AJGP-11-21-6229

24. Suk K jin, Mi L S. A Comparative Analysis on Current Status of Telemedicine Policy before and after COVID-19: Focused on France and Australia[J]. The Journal of Convergence Society and Public Policy, 2022, 16(3): 129-160. DOI: 10.37582/cspp.2022.16.3.129

25. seon J kwan. A Study on the Legislation of Telemedicine in France[J]. THE KOREAN SOCIETY OF LAW AND MEDICINE, 2022, 23(2): 141-169. DOI: 10.29291/kslm.2022.23.2.141

26. Gerke S, Stern A D, Minssen T. Germany’s digital health reforms in the COVID-19 era: lessons and opportunities for other countries[J]. npj Digital Medicine, Nature Publishing Group, 2020, 3(1): 1-6. PMID: 32685700

27. Cui WB, Zhang KK, Gu ST, et al. Research on "Internet +" medical service included in medical insurance reimbursement [J]. Chinese Hospitals, 2020(03), 24: 03. DOI: 10.19660/j.issn.1671-0592.2020.03.02 https://d.wanfangdata.com.cn/periodical/ChlQZXJpb2RpY2FsQ0hJTmV3UzIwMjQwNzA0EhF6aG9uZ2d5eTIwMjAwMzAwMhoIeW5tYXZzdHI%3D

28. Ma SQ, Cao Y. Relevant Research and Policy Suggestions on the Inclusion of "Internet Plus Medical" in Health Insurance Payment[J]. Modern Business Trade Industry, 2021(35), 42: 35. DOI: 10.19311/j.cnki.1672-3198.2021.35.045 https://d.wanfangdata.com.cn/periodical/ChlQZXJpb2RpY2FsQ0hJTmV3UzIwMjQwNzA0Eg94ZHNtZ3kyMDIxMzUwNDUaCHV3Mnlpc2Fh

29. Xiao GJ, Xu HF. Analysis and enlightenment of Medicare payment Internet medical service policy in America [J]. Soft Science of Health, 2022, 36(8): 90-96. DOI: 10.3969/j.issn.1003-2800.2022.08.018 https://d.wanfangdata.com.cn/periodical/ChlQZXJpb2RpY2FsQ0hJTmV3UzIwMjQwNzA0Eg53c3JreDIwMjIwODAxOBoING4zYmdsYzI%3D

30. Bogulski C A, Acharya M, Pro G. A State Profile of Disparities in Telehealth Utilization Among Medicare Beneficiaries: An Intersection Between Race/Ethnicity, Rurality, and Chronic Conditions-Arkansas, 2019[J]. Telehealth Journal and E-Health: The Official Journal of the American Telehealth Association, 2023. PMID: 37074340

31. Hall Dykgraaf S, Desborough J, de Toca L. “A decade’s worth of work in a matter of days”: The journey to telehealth for the whole population in Australia[J]. International Journal of Medical Informatics, 2021, 151. PMID:33984625

32. Raes S, Trybou J, Annemans L. How to Pay for Telemedicine: A Comparison of Ten Health Systems[J]. Health Systems and Reform, 2022, 8(1): 2116088. DOI:10.1080/23288604.2022.2116088

33. Fisk M, Livingstone A, Pit S W. Telehealth in the context of COVID-19: Changing perspectives in Australia, the United Kingdom, and the United States[J]. Journal of Medical Internet Research, 2020, 22(6). PMID: 32463377

34. Kim, J.S., & Lim, S.M. (2022). A Comparative Analysis on Current Status of Telemedicine Policy before and after COVID-19: Focused on France and Australia. The Journal of Convergence Society and Public Policy. DOI：10.37582/cspp.2022.16.3.129

35. Wilson FA, Rampa S, Trout KE, Stimpson JP. Reimbursements for telehealth services are likely to be lower than non-telehealth services in the United States. J Telemed Telecare. 2017;23(4):497-500. PMID: 27260264

36. Neufeld J D, Doarn C R, Aly R. State Policies Influence Medicare Telemedicine Utilization[J]. Telehealth Journal and E-Health: The Official Journal of the American Telehealth Association, 2016, 22(1): 70-74. PMID: 26218148

37. Khera N, Knoedler M, Meier S K. Payment and Coverage Parity for Virtual Care and In-Person Care: How Do We Get There?[J]. Telehealth Reports, 2023, 4(1): 100-108. DOI: 10.1089/tmr.2023.0014

38. Kinoshita S, Cortright K, Crawford A. Changes in telepsychiatry regulations during the COVID-19 pandemic: 17 countries and regions’ approaches to an evolving healthcare landscape[J]. Psychological medicine, 2022, 52(13): 2606-2613. DOI: 10.1017/S0033291720004584
