# Supplementary material for: Health Insurance Payment for Telehealth Services: Scoping Review and Narrative Synthesis
Source: J Med Internet Res. 2024 Dec 9;26:e56699. doi: 10.2196/56699 (PMC11668521; doi:10.2196/56699)
Supplement: Multimedia Appendix 4 [file jmir_v26i1e56699_app4.docx]

| Author/Year | Q 1 | Q 2 | Q 3 | Q 4 | Q 5 | Q 6 | Q 7 | Q 8 | Score | Classification  of Quality |
| --- | --- | --- | --- | --- | --- | --- | --- | --- | --- | --- |
| Li GY, Yao Q, Liu XJ, et al. (2023)[16] | 1 | 1 | 1 | 1 | 1 | 1 | 1 | 1 | 8 | High |
| Ding TT, Qian AB, Tan ZX (2022)[17] | 1 | 1 | 1 | 1 | 1 | 1 | 1 | 1 | 8 | High |
| Liang HM, Xu JJ, Peng Y, et al. (2020)[18] | 1 | 1 | 1 | 1 | 1 | 1 | 1 | 1 | 8 | High |
| Cui WB, Zhang KK, Gu ST, et al. (2020) [19] | 1 | 1 | 1 | 1 | 1 | 1 | 1 | 1 | 8 | High |
| Liu Y, Li Y, Jiang X, et al. (2021) [20] | 1 | 1 | 1 | 1 | 1 | 1 | 1 | 1 | 8 | High |
| Park S, Langellier BA, Burke RE (2021) [21] | 1 | 1 | 0 | 1 | 1 | 1 | 1 | 1 | 7 | High |
| Abbasi-Feinberg F (2020) [22] | 1 | 1 | 1 | 0 | 1 | 1 | 0 | 1 | 6 | Middle |
| Fisher K, Davey AR, Magin P (2022) [23] | 1 | 1 | 1 | 1 | 1 | 1 | 1 | 1 | 8 | High |
| Suk K jin, Mi LS (2022) [24] | 1 | 1 | 1 | 0 | 1 | 1 | 1 | 1 | 7 | High |
| Seon J kwan (2022) [25] | 1 | 1 | 1 | 0 | 1 | 1 | 1 | 1 | 7 | High |
| Gerke S, Stern AD, Minssen T (2020) [26] | 1 | 1 | 1 | 1 | 1 | 1 | 1 | 1 | 8 | High |
| Cui WB, Zhang KK, Gu ST, et al. (2020) [27] | 1 | 1 | 1 | 1 | 1 | 1 | 1 | 1 | 8 | High |
| Ma SQ, Cao Y (2021) [28] | 1 | 1 | 1 | 1 | 1 | 1 | 1 | 1 | 8 | High |
| Xiao GJ, Xu HF (2022) [29] | 1 | 1 | 1 | 1 | 1 | 1 | 1 | 1 | 8 | High |
| Bogulski CA, Acharya M, Pro G (2023) [30] | 1 | 1 | 0 | 1 | 1 | 1 | 1 | 1 | 7 | High |
| Hall Dykgraaf S, Desborough J, de Toca L (2021) [31] | 1 | 1 | 0 | 0 | 1 | 1 | 1 | 1 | 6 | Middle |
| Raes S, Trybou J, Annemans L (2022) [32] | 1 | 1 | 0 | 1 | 1 | 1 | 0 | 1 | 6 | Middle |
| Fisk M, Livingstone A, Pit SW (2020) [33] | 1 | 1 | 1 | 1 | 1 | 1 | 1 | 1 | 8 | High |
| Kim JS, Oh SH (2018) [34] | 1 | 1 | 1 | 0 | 1 | 1 | 1 | 1 | 7 | High |
| Wilson FA, Rampa S, Trout KE(2017) [35] | 1 | 1 | 1 | 1 | 1 | 1 | 1 | 1 | 8 | High |
| Neufeld JD, Doarn CR, Aly R (2016) [36] | 1 | 1 | 1 | 0 | 1 | 1 | 0 | 1 | 6 | Middle |
| Khera N, Knoedler M, Meier SK (2023) [37] | 1 | 1 | 1 | 1 | 1 | 1 | 0 | 1 | 7 | High |
| Kinoshita S, Cortright K, Crawford A (2022) [38] | 1 | 1 | 1 | 0 | 1 | 1 | 1 | 1 | 7 | High |

^a^Q1 = Was there a clear statement of the aims of the research?

^b^Q2 = Is a qualitative methodology appropriate?

^c^Q3 = Was the research design appropriate to address the aims of the research?

^d^Q4 = Are the evaluation metrics used in the study the most relevant?

^e^Q5 = Was the data collected in a way that addressed the research issue?

^f^Q6 = Was the data analysis sufficiently rigorous?

^g^Q7 = Is there a clear statement of findings?

^h^Q8 = Are the research limitations presented?

**Reference**

16. Li GY, Yao Q, Liu XJ, et al. Research on Medical Insurance Payment Policy of "Internet Plus" Medical Service in China [J]. Health Economics Research, 2023, 40(05): 43-46. https://d.wanfangdata.com.cn/periodical/wsjjyj202305011

17. Ding TT, Qian AB, Tan ZX. Comparative analysis of domestic and foreign Internet medical service pricing and medical insurance payment policies[J]. Chinese Hospitals, 2022, 26(9): 10-13. Doi:10.19660/j.issn.1671-0592.2022.9.03 https://d.wanfangdata.com.cn/periodical/ChlQZXJpb2RpY2FsQ0hJTmV3UzIwMjQwNzA0EhF6aG9uZ2d5eTIwMjIwOTAwMxoIN3hnM2RsdXY%3D

18. Liang HM, Xu JJ, Peng Y, et al. Analysis on the Core Issues of Pricing and Payment of "Internet+” Medical Services in China [J]. Chinese Health Economics, 2020(12), 39: 52-56. DOI: 10.7664/CHE20201212 https://d.wanfangdata.com.cn/periodical/ChlQZXJpb2RpY2FsQ0hJTmV3UzIwMjQwNzA0Eg96Z3dzamoyMDIwMTIwMTQaCGJhaGVsYWdz

19. Cui WB, Zhang KK, Gu ST, et al. Policy suggestions on inclusion of "internet +” medical services into medical insurance payment [J]. Chinese Health Resources, 2020, 23(2): 102-105+147. DOI: 10.3969/j.issn.1007-953X.2020.02.004 https://d.wanfangdata.com.cn/periodical/ChlQZXJpb2RpY2FsQ0hJTmV3UzIwMjQwNzA0Eg96Z3dzenkyMDIwMDIwMDQaCDc2a2FjaWE3

20. Liu Y, Li Y, Jiang X, et al. Analysis and enlightenment of health insurance payment policy for Internet medical services in the United States[J]. Chinese Journal of Hospital Administration, 2021, 37(8): 642-646. DOI: 10.3760/cma.j.cn111325-20210303-00173 https://d.wanfangdata.com.cn/periodical/ChlQZXJpb2RpY2FsQ0hJTmV3UzIwMjQwNzA0Eg96aHl5Z2wyMDIxMDgwMDYaCHQ2M2Ezdnly

21. Park S, Langellier B A, Burke R E. Telehealth Benefits Offered by Medicare Advantage Plans in 2020[J]. Medical Care, 2021, 59(1): 53-57. DOI:10.1097/MLR.00000000000001408

22. Abbasi-Feinberg F. Telemedicine Coding and Reimbursement - Current and Future Trends [J]. Sleep Medicine Clinics, 2020, 15(3): 417-429. PMID:32762974

23. Fisher K, Davey A R, Magin P. Telehealth for Australian general practice: The present and the future[J]. Australian Journal of General Practice, 2022, 51(8): 626-629. DOI: 10.31128/AJGP-11-21-6229

24. Suk K jin, Mi L S. A Comparative Analysis on Current Status of Telemedicine Policy before and after COVID-19: Focused on France and Australia[J]. The Journal of Convergence Society and Public Policy, 2022, 16(3): 129-160. DOI: 10.37582/cspp.2022.16.3.129

25. seon J kwan. A Study on the Legislation of Telemedicine in France[J]. THE KOREAN SOCIETY OF LAW AND MEDICINE, 2022, 23(2): 141-169. DOI: 10.29291/kslm.2022.23.2.141

26. Gerke S, Stern A D, Minssen T. Germany’s digital health reforms in the COVID-19 era: lessons and opportunities for other countries[J]. npj Digital Medicine, Nature Publishing Group, 2020, 3(1): 1-6. PMID: 32685700

27. Cui WB, Zhang KK, Gu ST, et al. Research on "Internet +" medical service included in medical insurance reimbursement [J]. Chinese Hospitals, 2020(03), 24: 03. DOI: 10.19660/j.issn.1671-0592.2020.03.02 https://d.wanfangdata.com.cn/periodical/ChlQZXJpb2RpY2FsQ0hJTmV3UzIwMjQwNzA0EhF6aG9uZ2d5eTIwMjAwMzAwMhoIeW5tYXZzdHI%3D

28. Ma SQ, Cao Y. Relevant Research and Policy Suggestions on the Inclusion of "Internet Plus Medical" in Health Insurance Payment[J]. Modern Business Trade Industry, 2021(35), 42: 35. DOI: 10.19311/j.cnki.1672-3198.2021.35.045 https://d.wanfangdata.com.cn/periodical/ChlQZXJpb2RpY2FsQ0hJTmV3UzIwMjQwNzA0Eg94ZHNtZ3kyMDIxMzUwNDUaCHV3Mnlpc2Fh

29. Xiao GJ, Xu HF. Analysis and enlightenment of Medicare payment Internet medical service policy in America [J]. Soft Science of Health, 2022, 36(8): 90-96. DOI: 10.3969/j.issn.1003-2800.2022.08.018 https://d.wanfangdata.com.cn/periodical/ChlQZXJpb2RpY2FsQ0hJTmV3UzIwMjQwNzA0Eg53c3JreDIwMjIwODAxOBoING4zYmdsYzI%3D

30. Bogulski C A, Acharya M, Pro G. A State Profile of Disparities in Telehealth Utilization Among Medicare Beneficiaries: An Intersection Between Race/Ethnicity, Rurality, and Chronic Conditions-Arkansas, 2019[J]. Telehealth Journal and E-Health: The Official Journal of the American Telehealth Association, 2023. PMID: 37074340

31. Hall Dykgraaf S, Desborough J, de Toca L. “A decade’s worth of work in a matter of days”: The journey to telehealth for the whole population in Australia[J]. International Journal of Medical Informatics, 2021, 151. PMID:33984625

32. Raes S, Trybou J, Annemans L. How to Pay for Telemedicine: A Comparison of Ten Health Systems[J]. Health Systems and Reform, 2022, 8(1): 2116088. DOI:10.1080/23288604.2022.2116088

33. Fisk M, Livingstone A, Pit S W. Telehealth in the context of COVID-19: Changing perspectives in Australia, the United Kingdom, and the United States[J]. Journal of Medical Internet Research, 2020, 22(6). PMID: 32463377

34. Kim, J.S., & Lim, S.M. (2022). A Comparative Analysis on Current Status of Telemedicine Policy before and after COVID-19: Focused on France and Australia. The Journal of Convergence Society and Public Policy. DOI：10.37582/cspp.2022.16.3.129

35. Wilson FA, Rampa S, Trout KE, Stimpson JP. Reimbursements for telehealth services are likely to be lower than non-telehealth services in the United States. J Telemed Telecare. 2017;23(4):497-500. PMID: 27260264

36. Neufeld J D, Doarn C R, Aly R. State Policies Influence Medicare Telemedicine Utilization[J]. Telehealth Journal and E-Health: The Official Journal of the American Telehealth Association, 2016, 22(1): 70-74. PMID: 26218148

37. Khera N, Knoedler M, Meier S K. Payment and Coverage Parity for Virtual Care and In-Person Care: How Do We Get There?[J]. Telehealth Reports, 2023, 4(1): 100-108. DOI: 10.1089/tmr.2023.0014

38. Kinoshita S, Cortright K, Crawford A. Changes in telepsychiatry regulations during the COVID-19 pandemic: 17 countries and regions’ approaches to an evolving healthcare landscape[J]. Psychological medicine, 2022, 52(13): 2606-2613. DOI: 10.1017/S0033291720004584
